# Blocking ADP-ribosylation expands the anti-mycobacterial spectrum of rifamycins

**DOI:** 10.1128/spectrum.01900-23

**Published:** 2023-09-08

**Authors:** Uday S. Ganapathy, Tian Lan, Véronique Dartois, Courtney C. Aldrich, Thomas Dick

**Affiliations:** 1 Center for Discovery and Innovation, Hackensack Meridian Health, Nutley, New Jersey, USA; 2 Department of Medicinal Chemistry, College of Pharmacy, University of Minnesota, Minneapolis, Minnesota, USA; 3 Department of Medical Sciences, Hackensack Meridian School of Medicine, Nutley, New Jersey, USA; 4 Department of Microbiology and Immunology, Georgetown University, Washington, DC, USA; CNRS-University of Toulouse, Toulouse, France

**Keywords:** rifamycins, NTM, non-tuberculous mycobacteria, ADP-ribosylation, *M. abscessus*, *M. chelonae*, *M. fortuitum*, *M. xenopi*, *M. simiae*

## Abstract

The clinical utility of rifamycins against non-tuberculous mycobacterial (NTM) disease is limited by intrinsic drug resistance achieved by ADP-ribosyltransferase Arr. By blocking the site of ribosylation, we recently optimized a series of analogs with substantially improved potency against *Mycobacterium abscessus*. Here, we show that a representative member of this series is significantly more potent than rifabutin against major NTM pathogens expressing Arr, providing a powerful medicinal chemistry approach to expand the antimycobacterial spectrum of rifamycins.

**IMPORTANCE:**

Lung disease caused by a range of different species of non-tuberculous mycobacteria (NTM) is difficult to cure. The rifamycins are very active against *Mycobacterium tuberculosis,* which causes tuberculosis (TB), but inactive against many NTM species. Previously, we showed that the natural resistance of the NTM *Mycobacterium abscessus* to rifamycins is due to enzymatic inactivation of the drug by the bacterium. We generated chemically modified versions of rifamycins that prevent inactivation by the bacterium and thus become highly active against *M. abscessus.* Here, we show that such a chemically modified rifamycin is also highly active against several additional NTM species that harbor the rifamycin inactivating enzyme found in *M. abscessus*, including *M. chelonae*, *M. fortuitum*, and *M. simiae*. This finding expands the potential therapeutic utility of our novel rifamycins to include several currently difficult-to-cure NTM lung disease pathogens beyond *M. abscessus.*

## INTRODUCTION

The incidence of non-tuberculous mycobacterial pulmonary disease (NTM-PD) is increasing globally (1 – 3). These infections are caused by a diverse range of opportunistic mycobacterial species, which can be categorized into rapidly growing and slowly growing NTM. Among the rapidly growing NTM, *Mycobacterium abscessus* complex is the most common pathogen and consists of three subspecies (subsp. *abscessus*, subsp. *bolletii*, and subsp. *massiliense*) (4, 5). Other rapidly growing NTMs that are clinically significant include *M. chelonae* and *M. fortuitum* (6, 7). Among the slowly growing NTM, *M. avium* complex (MAC) is responsible for most pulmonary infections, with three MAC species being most frequently involved: *M. avium*, *M. intracellulare*, and *M. chimaera* (4, 8, 9). Other clinically relevant members of the slowly growing NTMs include *Mycobacterium kansasii*, *Mycobacterium szulgai*, *Mycobacterium xenopi*, and *Mycobacterium simiae* (4, 10 – 12). *M. xenopi* infections have the highest mortality rates of any NTM (13, 14), while *M. simiae* displays the most severe intrinsic drug resistance (12). Treatment for NTM-PD relies heavily on macrolide-based multidrug regimens, combining azithromycin or clarithromycin with additional drugs tailored to the NTM species involved (4, 15). These drug regimens, however, are largely suboptimal, and treatment outcomes remain poor. The average cure rates of *M. abscessus* complex and MAC pulmonary infections are 50% and 60%, respectively (16, 17). More efficacious drugs are urgently needed to improve NTM-PD regimens.

Rifamycins are a cornerstone of tuberculosis (TB) chemotherapy. By targeting the β-subunit of the mycobacterial RNA polymerase (RpoB), these drugs have bactericidal activity against both replicating and non-replicating *Mycobacterium tuberculosis* (18 – 20) and kill this bacterium within macrophages (21) and caseous lesions (22, 23). These properties enable exceptional sterilizing activity in the clinic, where the addition of rifampicin to the TB drug regimen reduced the relapse rate and shortened treatment time to 6 months (24). Despite this track record, rifamycins have limited clinical use against NTM-PD. They are not recommended to treat infections by any rapidly growing NTM due to intrinsic rifamycin resistance (Table 1) (4, 15). Although they are used to treat most slowly growing NTMs, with the exception of *M. simiae*, which displays high intrinsic rifamycin resistance (Table 1) (12, 15), clinical confidence in rifamycin treatment of slowly growing NTMs is only firmly established for *M. kansasii* (4). In addition, MAC species display a broad rifamycin MIC distribution that exceeds clinical breakpoints and likely contributes to variable treatment outcomes (25 – 29). Rifamycin resistance in these MAC isolates is not linked to acquired RpoB mutations, suggesting the presence of intrinsic rifamycin resistance mechanisms (30) and preventing this critical sterilizing TB drug class from similarly contributing to NTM-PD cure.

**TABLE 1:** Broad spectrum anti-mycobacterial profiling of compound RFB-5m

			MIC (µM)^*a*^				
Strain	Rifamycins used clinically? (4, 15, 31)	Arr homolog present? (32)	CLR^*b*^	RIF	RFB	RFB-5m	Fold improvement (RFB-5m vs RFB)
Rapidly growing NTM							
*M. abscessus* complex							
*M. abscessus* subsp. *abscessus* ATCC 19977	No	Yes	1.5	9.5	1.1	0.02	55
*M. abscessus* subsp. *bolletii* CCUG 50184T	No	Yes	4.3	28	2.2	0.07	31
*M. abscessus* subsp. *massiliense* CCUG 48898T	No	Yes	1.1	13	0.6	0.05	12
*M. chelonae* ATCC 35752	No	Yes	0.1	1.4	0.6	0.02	30
*M. fortuitum* ATCC 6841	No	Yes	3.2	6.7	1.2	0.02	60
Slowly growing NTM							
*M. avium* complex							
*M. avium* subsp. *hominisuis* MAC109	Yes	No	0.5	0.04	0.03	0.04	0.8
*M. intracellulare* ATCC 13950	Yes	No	0.4	0.06	0.05	0.06	0.8
*M. chimaera* CCUG 50989T	Yes	No	0.8	0.07	0.05	0.06	0.8
*M. kansasii* ATCC 12478	Yes	No	0.3	0.3	0.008	0.008	1.0
*M. szulgai* ATCC 35799	Yes	No	0.3	0.06	0.01	0.006	1.7
*M. xenopi* ATCC 19250	Yes	Yes	0.06	0.04	0.02	0.005	4.0
*M. simiae* ATCC 25275	No	Yes	12	>100	19	0.2	95

^*a*^ MIC values are the mean of two independent experiments.

^*b*^ CLR, clarithromycin; RIF, rifampicin; and RFB, rifabutin.

In recent years, a dual mechanism of intrinsic rifamycin resistance has been identified in *M. abscessus* subsp. *abscessus*. First, bacterial monooxygenases can oxidize rifamycins that contain a naphthohydroquinone core (e.g., rifampicin), but this enzymatic oxidation is prevented in rifamycins with a naphthoquinone core (e.g., rifabutin) (33, 34), explaining why rifabutin is 5–10 times more potent than rifampicin against *M. abscessus* subsp. *abscessus* (33, 35). Second, the ADP-ribosyltransferase Arr*_Mab_* can conjugate an ADP-ribose to the C23 hydroxyl group of rifamycins, including rifampicin and rifabutin (33, 36, 37). This activity shifts the MIC of rifabutin against *M. abscessus* subsp. *abscessus* by ~50-fold, preventing this rifamycin from achieving nanomolar potency, as it does against *M. tuberculosis* (33). Thus, ADP-ribosylation represents the major mechanism of intrinsic rifamycin resistance in this subspecies.

Recently, we reported 25-*O*-desacetyl-25-*O*-nicotinoylrifabutin (RFB-5m), a member of a novel series of rifabutin analogs designed to overcome *M. abscessus* intrinsic rifamycin resistance (38). By retaining the same naphthoquinone core as rifabutin, RFB-5m can prevent enzymatic oxidation. Critically, RFB-5m’s novel C25 group blocks ADP-ribosylation by Arr*_Mab_* (38). As a result, RFB-5m is 50 times more potent against *M. abscessus* subsp. *abscessus* than rifabutin with an MIC of 24 nM (38). Therefore, the activity of rifamycins against *M. abscessus* subsp. *abscessus* can be dramatically improved with chemical modifications that block Arr-mediated resistance.

## RESULTS AND DICUSSION

**Fig 1:**
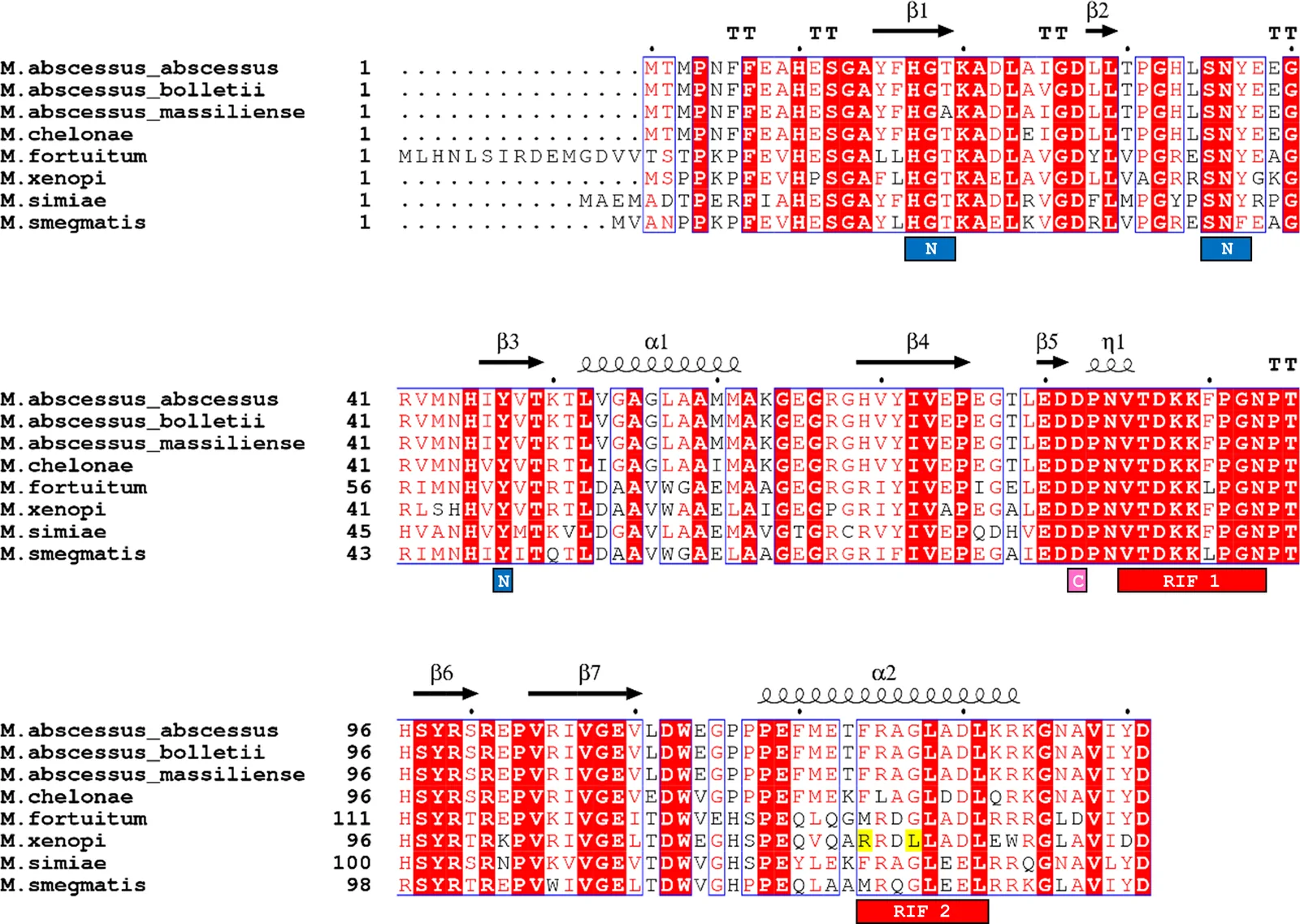
Conservation of Arr across NTM species. The amino acid sequences of Arr homologs from selected NTM species (Table 1) and *M. smegmatis* (Arr*_Msm_*) were aligned using Clustal Omega (41). The alignment was displayed with ESPript 3.0 (42) to show conserved residues (in red) and strict identity (in white on red background). Arr*_Msm_* is the prototypical Arr that confers rifamycin resistance (MICs of RIF, RFB, and RFB-5m against *M. smegmatis* ATCC 700084 are 18 µM, 2.5 µM, and 0.16 µM, respectively) and for which the 3D structure has been determined (PDB 2HW2) (40). Based on this structure, NAD^+^ binding sites (blue boxes labeled with N), rifampicin binding sites (red boxes labeled RIF 1 and RIF 2), and the D84 catalytic residue (pink box labeled with C)—required for stabilization of the oxocarbenium ion in the transition state—were identified (40). Structural elements are indicated above the sequence alignment: TT, beta turn; β, beta strand; α, alpha helix; η, 3_10_ helix. NTM Arr homologs showed high levels of conservation of these motifs as well as across the entire sequence length. For each Arr homolog, the identity and similarity to Arr*_Msm_* were determined: *M. abscessus* subsp. *abscessus* ATCC 19977, 65.9% and 76.8%; *M. abscessus* subsp. *bolletii* CCUG 50184T, 66.7% and 76.8%; *M. abscessus* subsp. *massiliense* CCUG 48898T, 65.2% and 76.1%; *M. chelonae* ATCC 35752, 65.2% and 78.3%; *M. fortuitum* ATCC 6841, 79.7% and 88.8%; *M. xenopi* ATCC 19250, 72.1% and 82.1%; *M. simiae* ATCC 25275, 62.7% and 77.5%. In the sequence of *M. xenopi* Arr, we identified two residues in rifampicin binding site 2 (R125 and L128, yellow highlights) with differential physicochemical properties compared to those found in other NTM (positively charged R125 vs nonpolar M/F residues; branched-chain, aliphatic L128 vs compact G residues).

In an extensive genomics study (32), the distribution of Arr homologs across the bacteria domain was determined. All rapidly growing NTM were found to harbor an Arr homolog, including all *M. abscessus* complex members, *M. chelonae*, and *M. fortuitum* (Table 1) (32). While Arr was absent from most slowly growing NTM, homologs were identified in *M. xenopi* and *M. simiae* (Table 1) (32). To explore whether these putative Arr homologs represent functional rifamycin ADP-ribosyltransferases, we aligned their protein sequences along with those of two experimentally validated Arr proteins: Arr*_Mab_* (36) and Arr*_Msm_*, the prototypical Arr from the non-pathogenic model organism *M. smegmatis* for which the 3D structure has been determined (Fig. 1) (39, 40). A high level of conservation was observed across the full length of all Arr sequences analyzed, including all motifs associated with rifamycin ADP-ribosyltransferase activity (Fig. 1) (40), suggesting that the putative Arr homologs are likely functional ADP-ribosyltransferases that may drive intrinsic rifamycin resistance in their respective NTM species.

Given this high degree of Arr conservation across NTM species, we reasoned that RFB-5m would show enhanced activity against NTM species with an Arr homolog. To test this hypothesis, we determined the MIC of RFB-5m and rifabutin against a panel of Arr-positive and Arr-negative species representing a range of rapidly and slowly growing NTM (Table 1) in Middlebrook 7H9 broth and 96-well plates as previously described (33). Plates were incubated at 37°C for either 3 days (for rapidly growing NTM) or 5 days (for slowly growing NTM). As expected, RFB-5m showed improved and comparable potency over rifabutin against all Arr-harboring NTM tested (Table 1), including all members of the *M. abscessus* complex (subsp. *abscessus*, *bolletii*, and *massiliense*) (Table 1) and the other clinically relevant rapidly growing NTM: *M. chelonae* and *M. fortuitum* (Table 1). In addition, RFB-5m showed improved activity against *M. xenopi* and *M. simiae*, two members of the slowly growing NTM that harbor Arr homologs (Table 1). Remarkably, RFB-5m was 95 times more active than rifabutin against *M. simiae* and achieved a sub-micromolar MIC (Table 1), effectively transforming the rifamycins from an inactive drug class into one with therapeutic potential.

Against *M. xenopi*, RFB-5m was only four times more potent than rifabutin—a noticeably smaller potency shift than those we observed with other Arr-harboring NTM—which appears to be due to a higher potency of the parent rifamycins rather than a lower potency of RFB-5m (Table 1). We hypothesized that *M. xenopi* Arr has reduced rifamycin ADP-ribosyltransferase activity, resulting in weaker rifamycin resistance. Consistent with this hypothesis, we identified two amino acid residues in the rifampicin binding site 2 of *M. xenopi* Arr that differ from those found in other NTM Arr homologs (Fig. 1). Both differences lead to non-conservative amino acid substitutions (Fig. 1), which could alter the configuration of rifampicin binding site 2, thereby reducing the enzyme’s rifamycin ADP-ribosylation activity.

In conclusion, we found that the rifabutin analog RFB-5m overcomes intrinsic rifamycin resistance in a range of NTM species. RFB-5m had strongly enhanced potency against all members of the *M. abscessus* complex, other clinically relevant rapidly growing NTM (*M. chelonae* and *M. fortuitum*), and the slowly growing NTM *M. simiae*, all encoding Arr and linking RFB-5m’s enhanced activity to its ability to block ADP-ribosylation (38). Therefore, our data demonstrate that ADP-ribosylation drives intrinsic rifamycin resistance in several NTM besides *M. abscessus* subsp. *abscessus*. MIC testing with a larger set of NTM isolates will be needed to fully characterize the prevalence of Arr-mediated rifamycin resistance. Critically, our results indicate that Arr-tolerant rifamycins in development for *M. abscessus* lung disease (38) can be applied to other Arr-positive NTMs. C25-modified rifamycins like RFB-5m now present an exciting opportunity to expand the anti-mycobacterial spectrum of rifamycins.

## MATERIALS AND METHODS

The amino acid sequences of Arr homologs from selected mycobacterial species were aligned using Clustal Omega (41). The alignment was displayed with ESPript 3.0 (42) using the percentage equivalent similarity score scheme set at 0.6. Sequence database IDs are as follows: *M. abscessus* subsp. *abscessus* ATCC 19977, CAM60689.1; *M. abscessus* subsp. *bolletii* CCUG 50184T, AMU23538.1; *M. abscessus* subsp. *massiliense* CCUG 48898T, EIV69432.1; *M. chelonae* ATCC 35752, ANA96794.1; *M. fortuitum* ATCC 6841, BDE01014.1; *M. xenopi* ATCC 19250, BBU20527.1; *M. simiae* ATCC 25275, BBX43478.1; and *M. smegmatis*, 2HW2_A.

All mycobacterial strains were obtained from culture collections as indicated in Table 1 except *M. avium* subsp. *hominissuis* MAC109, which was provided by Petros C. Karakousis (Johns Hopkins University). MICs were determined by the broth dilution method using Middlebrook 7H9 broth and OD_600_ as readout as described previously (33). Dose response curves were generated, and drug concentrations inhibiting 90% of bacterial growth were defined as MIC (33).
